# Prospective comparison of various radiological response criteria and pathological response to preoperative chemotherapy and survival in operable high-grade soft tissue sarcomas in the Japan Clinical Oncology Group study JCOG0304

**DOI:** 10.1186/s12957-018-1462-y

**Published:** 2018-08-10

**Authors:** Kazuhiro Tanaka, Gakuto Ogawa, Junki Mizusawa, Norifumi Naka, Akira Kawai, Mitsuru Takahashi, Toru Hiruma, Yoshihiro Matsumoto, Hiroyuki Tsuchiya, Robert Nakayama, Hiroshi Hatano, Makoto Emori, Masami Hosaka, Yukihiro Yoshida, Junya Toguchida, Satoshi Abe, Kunihiro Asanuma, Ryohei Yokoyama, Hiroaki Hiraga, Tsukasa Yonemoto, Takeshi Morii, Seiichi Matsumoto, Akihito Nagano, Hideki Yoshikawa, Haruhiko Fukuda, Toshifumi Ozaki, Yukihide Iwamoto

**Affiliations:** 1 Department of Orthopaedic Surgery, Oita University, Idaigaoka 1-1, Hasama, Yufu City, Oita 879-5593 Japan; 2 JCOG Data Center, National Cancer Center Hospital, Tokyo, 104-0045 Japan; 3 Musculoskeletal Oncology Service, Osaka International Cancer Institute, Osaka, 541-8567 Japan; 4 Department of Orthopaedic Surgery, National Cancer Center, Tokyo, 104-0045 Japan; 5 Department of Orthopaedic Surgery, Shizuoka Cancer Center, Shizuoka, 411-0934 Japan; 6 Department of Orthopaedic Surgery, Kanagawa Cancer Center, Kanagawa, 241-0815 Japan; 7 Department of Orthopaedic Surgery, Kyushu University, Fukuoka, 812-8582 Japan; 8 Department of Orthopaedic Surgery, Kanazawa University, Ishikawa, 920-8641 Japan; 9 Department of Orthopaedic Surgery, Keio University, Tokyo, 160-0016 Japan; 10 Department of Orthopaedic Surgery, Niigata Cancer Center Hospital, Niigata, 951-8133 Japan; 11 Department of Orthopaedic Surgery, Sapporo Medical University, Sapporo, 060-8556 Japan; 12 Department of Orthopaedic Surgery, Tohoku University, Sendai, 980-8575 Japan; 13 Department of Orthopaedic Surgery, Nihon University, Tokyo, 173-8610 Japan; 14 Department of Orthopaedic Surgery, Kyoto University, Kyoto, 606-8501 Japan; 15 Department of Orthopaedic Surgery, Teikyo University, Tokyo, 173-8606 Japan; 16 Department of Orthopaedic Surgery, Mie University, Mie, 514-8507 Japan; 17 Department of Orthopaedic Surgery, National Kyushu Cancer Center, Fukuoka, 811-1395 Japan; 18 Department of Orthopaedic Surgery, Hokkaido Cancer Center, Sapporo, 003-0804 Japan; 19 Department of Orthopaedic Surgery, Chiba Cancer Center, Chiba, 260-8717 Japan; 20 Department of Orthopaedic Surgery, Kyorin University, Tokyo, 181-8611 Japan; 21 Department of Orthopaedic Surgery, Cancer Institute Hospital, Tokyo, 135-8550 Japan; 22 Department of Orthopaedic Surgery, Gifu University, Gifu, 501-1194 Japan; 23 Department of Orthopaedic Surgery, Osaka University, Osaka, 565-0871 Japan; 24 Department of Orthopaedic Surgery, Okayama University, Okayama, 700-0914 Japan; 25 Kyushu Risai Hospital, Kitakyushu, 800-0296 Japan

**Keywords:** Soft tissue sarcoma, Preoperative chemotherapy, Radiological response criteria, Pathological response, Survival

## Abstract

**Background:**

Soft tissue sarcomas (STS) are rare malignant tumors. The efficacy of preoperative chemotherapy for STS is evaluated using various tumor size-based radiological response criteria. However, it is still unclear which set of criteria would show the best association with pathological response and survival of the patients with STS.

**Methods:**

We compared radiological responses to preoperative chemotherapy for operable STS by the Response Evaluation Criteria in Solid Tumors (RECIST), modified RECIST, World Health Organization criteria, Japanese Orthopaedic Association criteria, and modified Choi criteria and analyzed the association with pathological response and survival using the data from the Japan Clinical Oncology Group (JCOG) study JCOG0304, a phase II clinical trial evaluating the efficacy of perioperative chemotherapy for STS in the extremities.

**Results:**

Seventy eligible patients in JCOG0304 were analyzed. The results demonstrated that none of the size-based radiological response criteria showed significant association with pathological response to preoperative chemotherapy for STS. The difference between overall survival of the patients assessed as partial response and stable disease/progressive disease by RECIST was not significant (hazard ratio 1.37, *p* = 0.63), and calculated C-index was 0.50. All other response criteria also could not exhibit significant association between radiological responses and survival.

**Conclusion:**

In the present study, none of the radiological response criteria analyzed demonstrated association of response to preoperative chemotherapy with pathological response or survival of the patients with operable STS. Further prospective investigation is required to develop criteria to evaluate not only tumor shrinkage but biological effects of preoperative chemotherapy for the patients with localized STS.

**Trial registration:**

UMIN Clinical Trials Registry C000000096. Registered 30 August, 2005 (retrospectively registered).

## Background

Soft tissue sarcomas (STS) are rare malignant tumors accounting for approximately 1% of all malignancies in the USA [1]. According to the Japanese Orthopaedic Association (JOA) Soft Tissue Tumor Registry in Japan, 1529 STS patients were registered in 2015 [2].

It has been reported that preoperative chemotherapy is effective for operable, high-grade STS [3–5]. The standard chemotherapy regimen for STS is doxorubicin (DOX) and/or ifosfamide (IFM) [6–8]. DOX has been a key drug for many years in the treatment of STS and its response rate for STS has been reported to be approximately 25%, whereas IFM is another key drug in the chemotherapy for STS with response rate of approximately 30% [6, 7]. The responses to preoperative chemotherapy for operable STS have been evaluated using tumor size-based radiological response criteria such as the World Health Organization (WHO) [9], Response Evaluation Criteria in Solid Tumors (RECIST) [10], JOA [11], and Choi [12]. However, the surrogacy of the radiological responses to chemotherapy for survival of the patients with operable STS is still controversial.

Another modality to assess the efficacy of preoperative chemotherapy for STS is histological evaluation of tumor necrosis using the resected specimen after surgery. The pathological response to chemotherapy is thought to have better correlation with survival of the patients with STS than radiological responses [13–15]. However, the limitation of use of histological evaluation is that the information is available only after the surgical resection of the tumor. If the assessment of efficacy on survival after preoperative chemotherapy for STS is possible before surgery using radiological response evaluation, it is very useful for the planning of surgical margin and the decision of additional adjuvant treatment including radiotherapy before surgery.

Moreover, the radiological response is an important outcome since response rate to chemotherapy is often considered as the primary endpoint in phase II clinical trials. Especially in clinical trials in which many institutions participate, a modality to evaluate the response might be ideal if it is simple, quick, and objective. The response evaluation by changes in tumor size on the radiological image is much suitable for those conditions. However, it is still unclear which radiological response criteria exhibit the most reliable association with pathological response and/or survival of the patients with STS.

Comparison of WHO and RECIST and RECIST and Choi criteria in the evaluation of response to chemotherapy for advanced STS has been reported [16, 17]. For operable STS, the comparison of radiological criteria in local radiotherapy, hyperthermia, and perfusion therapy were reported [18–20]. However, there is no study prospectively comparing various size-based response criteria in the preoperative chemotherapy and analyzing association with both pathological response and survival of the patients with operable STS.

In the present study, we therefore conducted comparisons of the radiological responses to preoperative chemotherapy for operable STS assessed by RECIST, modified RECIST, WHO, JOA, and modified Choi criteria to elucidate the association with pathological response and survival using the data from JCOG0304, a phase II clinical trial evaluating the efficacy of perioperative chemotherapy consisting of DOX and IFM for STS in the extremities [5, 21].

## Methods

### Ethical statement

This ancillary study plan was included in the JCOG0304 protocol, and it was approved by the Clinical Trial Review Committee of JCOG and by the Institutional Review Boards of each of the 27 participating institutes. The written informed consents were obtained from all of the patients before entry to JCOG0304.

### Patients

JCOG0304 was conducted by the Bone and Soft Tissue Tumor Study Group (BSTTSG) of the Japan Clinical Oncology Group (JCOG) [5, 21]. The trial has been registered with the UMIN Clinical Trials Registry [http://www.umin.ac.jp/ctr/index.htm], and the registration number is C000000096. In brief, the main inclusion criteria of the trial were as follows: (1) Histologically proven STS using biopsy specimens diagnosed as synovial sarcoma, liposarcoma, leiomyosarcoma, undifferentiated pleomorphic sarcoma, undifferentiated sarcoma, fibrosarcoma, or pleomorphic rhabdomyosarcoma; (2) Federation Nationale des Centers de Lutte Contre le Cancer (FNCLCC) grading system grade 2 or 3; (3) American Joint Committee on Cancer staging (6th edition) stage III (T2bN0M0); (4) operable tumor localized in the extremities; (5) age ≥ 20 and ≤ 70 years; (6) Eastern Cooperative Oncology Group (ECOG) performance status 0 or 1.

Preoperative chemotherapy using DOX (30 mg/m^2^/day × 2 days) and IFM (2 g/m^2^/day × 5 days) was conducted for three courses with 3-week intervals. Thereafter, surgical resection of the tumor was performed within 5 weeks from the first day of the last course of preoperative chemotherapy, and the resected tumor was subjected to the assessment of histological response. After the surgery, postoperative chemotherapy using DOX (30 mg/m^2^/day × 2 days) and IFM (2 g/m^2^/day × 5 days) was performed for two courses every 3 weeks. The details of the surgery were reported elsewhere [5, 21].

### Evaluation of radiological response

The radiological response to chemotherapy was assessed after the last course of preoperative chemotherapy using magnetic resonance imaging (MRI) by changes in the tumor size according to RECIST, modified RECIST, WHO, JOA, and modified Choi criteria (Table 1). MRI of the tumor was conducted within 28 days before enrollment of the patient as the baseline evaluation. After preoperative chemotherapy, MRI of the tumor was conducted again between 14 and 28 days after the first day of the last course of the chemotherapy for the response evaluation. Briefly, in RECIST criteria, the response was measured using the longitudinal diameter of the cross section of the lesion and was classified into four groups: complete response (CR), no residual tumor; partial response (PR), 30% or greater decrease; stable disease (SD), no significant change (less than 30% decrease or less than 20% increase); progressive disease (PD), 20% or greater increase [10].

**Table 1:** Size-based criteria for response to chemotherapy

Criteria	RECIST	Modified RECIST	WHO	JOA	Modified Choi
Measurement	1 direction	2 directions	2 directions	1 and 2 directions	1 direction
CR	Disappear	Disappear	Disappear	Disappear	Disappear
PR	≥ 30% decrease	≥ 50% decrease	≥ 50% decrease	1d ≥ 30% decrease or 2d ≥ 50% decrease	≥ 10% decrease
MR	–	–	–	2d 25–50%	–
SD	non PR and PD	non PR and PD	non PR and PD	non PR and PD	non PR and PD
PD	≥ 20% increase	≥ 44% increase	≥ 25% increase	1d ≥ 10% increase or 2d ≥ 25% increase	≥ 10% increase

*CR* complete response, *PR* partial response, *MR* minor response, *SD* stable disease, *PD* progressive disease, *1d* one direction, *2d* two directions

In modified RECIST criteria, which was made specific to JCOG0304, the product of the largest perpendicular diameters of the cross section of the lesion was calculated, and the responses were assessed as follows: CR, no residual tumor; PR, 50% or greater decrease; SD, no significant change (less than 50% decrease or less than 44% increase); PD, 44% or greater increase [5].

In WHO criteria, the product of the largest perpendicular diameters of the cross section of the lesion was calculated, and the responses were assessed as follows: CR, no residual tumor; PR, 50% or greater decrease; SD, no significant change (less than 50% decrease or less than 25% increase); PD, 25% or greater increase [9].

In JOA criteria, the longitudinal diameter or the product of the largest perpendicular diameters of the cross section of the lesion was calculated, and the responses were defined as follows: CR, no residual tumor; PR, 30% or greater decrease (one direction) or 50% or greater decrease (two directions); minor response (MR), greater than 25% decrease or less than 50% decrease (two directions); SD, no significant change (less than 30% decrease or less than 10% increase in one direction, or less than 25% decrease or less than 25% increase in two directions); PD, 10% or greater increase (one direction) or 25% or greater decrease (two directions) [11].

In modified Choi criteria, the longitudinal diameter of the cross section of the lesion was measured, and the responses were classified as follows: CR, no residual tumor; PR, 10% or greater decrease; SD, no significant change (less than 10% decrease or less than 10% increase); PD, 10% or greater increase [12]. Since the present study focused only on the size change of STS, computed tomography (CT) density was not considered in the modified Choi criteria.

The radiological response was assessed as positive in the cases of CR and PR in those criteria except for JOA and in the cases of CR, PR, and MR for JOA.

### Evaluation of pathological response

The pathological response to preoperative chemotherapy using the surgical specimen was also evaluated. The pathological response (tumor necrosis) was defined as follows: grade 0, necrosis less than 50% of the surgical specimen; grade 1, 50–90% necrosis; grade 2, greater than 90% necrosis; grade 3, no viable tumor cells. The pathological response was assessed as positive in the cases of grade 2 and 3.

### Evaluation of survivals

The definition of progression free survival (PFS) and overall survival (OS) were previously described [5, 21]: PFS, time period from the day of surgery until the day of the first evidence of disease progression or death; OS, time period from the day of surgery until the day of death. The date of data cut-off for survival was November 4, 2015.

### Statistical analysis

Sensitivity, which was defined as a proportion of radiological response (CR, PR, MR) of tumors among those pathological response (grades 2 and 3), and specificity, which was defined as a proportion of radiological non-response (SD, PD) of tumors among those pathological non-response (grades 0 and 1) were calculated.

As a measure of concordance between radiological response and survival, concordance index (C-index) was calculated by Harrell’s method [22].

The PFS and OS were estimated using the Kaplan-Meier method. *p* values and hazard ratios (HRs) were calculated by the log-rank test and Cox proportional hazard regression model. Differences were considered significant when *p* values were < 0.05. Statistical analysis was performed using SAS version 9.4 (SAS Institute, Cary, NC).

## Results

### Response to preoperative chemotherapy

**Fig. 1:**
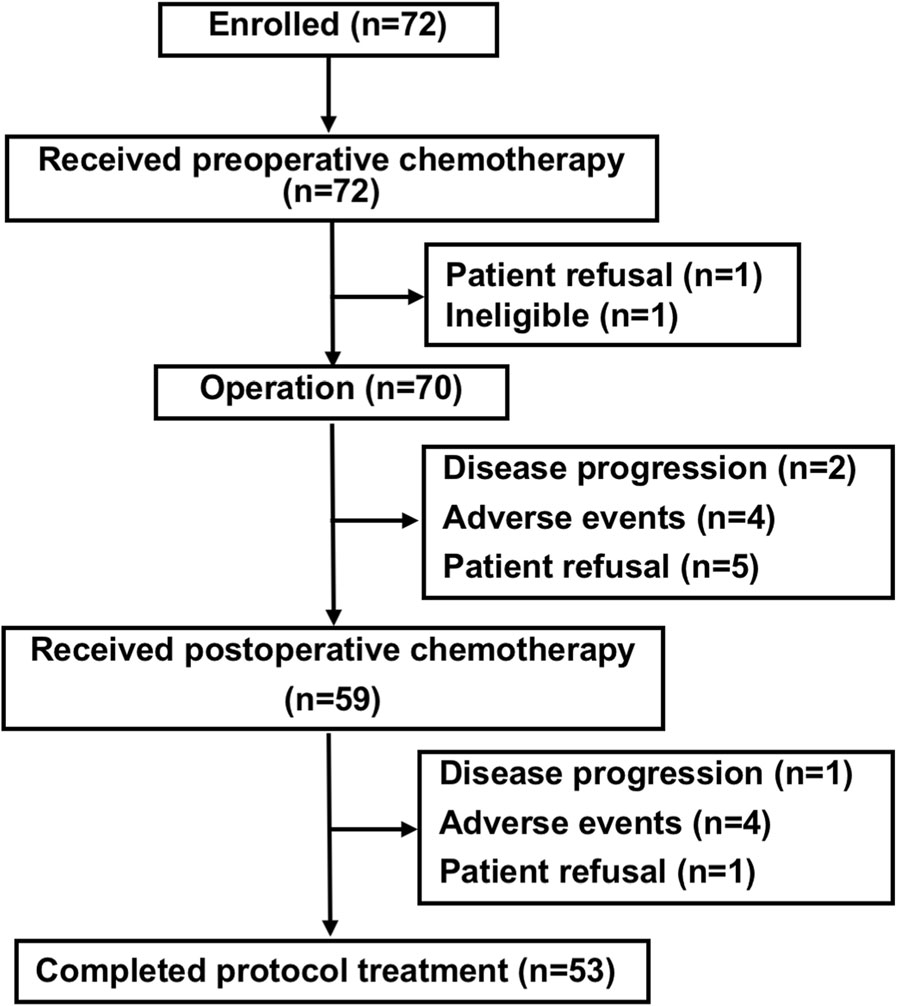
Patients flow diagram of JCOG0304

A total of 72 patients who suffered from operable high-grade STS in the extremities were enrolled in the JCOG0304 trial (Fig. 1). Details of the patient characteristics were reported previously [5]. Briefly, 70 out of 72 patients were eligible and assessed for radiological response to preoperative chemotherapy. The results of the size-based radiological response are summarized in Table 2. In RECIST evaluation, 12 PR, 53 SD, and 5 PD were observed, resulting in the response rate of 17.1% (95% CI, 9.2–28.0%). Using modified RECIST, 15 PR, 52 SD, and 3 PD were assessed, and response rate was 21.4% (95% CI, 12.5–32.9%). By WHO criteria, 15 PR, 49 SD, and 6 PD were found, and response rate was 21.4% (95% CI, 12.5–32.9%). The evaluation with JOA criteria demonstrated 17 PR, 8 MR, 36 SD, and 9 PD, and response rate including MR was 35.7% (95% CI, 24.6–48.1%). Finally, with modified Choi criteria, 27 PR, 35 SD, and 8 PD were observed, indicating response rate of 38.6% (95% CI, 27.2–51.0%).

**Table 2:** Radiological response to preoperative chemotherapy for STS

	RECIST	Modified RECIST	WHO	JOA	Modified Choi
CR	0	0	0	0	0
PR (MR)	12	15	15	17(8)	27
SD	53	52	49	36	35
PD	5	3	6	9	8
Response Rate	17.1%	21.4%	21.4%	35.7%	38.6%

*CR* complete response, *PR* partial response, *MR* minor response, *SD* stable disease, *PD* progressive disease

On the other hand, 68 patients were assessed for pathological response to the chemotherapy. The pathological evaluation demonstrated that 21 patients were assessed as grade 0, 27 as grade 1, 15 as grade 2, and 5 as grade 3. The proportion of pathological response to preoperative chemotherapy was found to be 28.6% (95% CI, 18.4–40.6%).

### Association between radiological response and pathological response

Table 3 shows the association of radiological responses and pathological response. The sensitivity and specificity with pathological response were 10.0% and 82.5% in RECIST, 20.0% and 77.8% in modified RECIST, 20.0% and 77.8% in WHO, 35.0% and 63.5% in JOA, and 35.0% and 60.3% in modified Choi, respectively.

**Table 3:** Correlation of radiological response with pathological response to preoperative chemotherapy for STS

		Pathological response (grade)						
		0	1	2	3	NE	Missing	Total
RECIST	PR	2	8	1	1	0	0	12
	SD	16	18	13	4	1	1	53
	PD	3	1	1	0	0	0	5
	Total	21	27	15	5	1	1	70
Modified RECIST	PR	1	10	3	1	0	0	15
	SD	18	16	12	4	1	1	52
	PD	2	1	0	0	0	0	3
	Total	21	27	15	5	1	1	70
WHO	PR	1	10	3	1	0	0	15
	SD	17	16	10	4	1	1	49
	PD	3	1	2	0	0	0	6
	Total	21	27	15	5	1	1	70
JOA	PR	2	10	3	2	0	0	17
	MR	2	4	2	0	0	0	8
	SD	11	12	8	3	1	1	36
	PD	6	1	2	0	0	0	9
	Total	21	27	15	5	1	1	70
Modified Choi	PR	4	16	5	2	0	0	27
	SD	11	10	9	3	1	1	35
	PD	6	1	1	0	0	0	8
	Total	21	27	15	5	1	1	70

*PR* partial response, *MR* minor response, *SD* stable disease, *PD* progressive disease, *NE* Not evaluated

### Association between pathological response and survival

The median follow-up period for all surviving patients in the present study was 8.7 years. At the data cut-off, 56 patients were alive and 14 patients had died. The probabilities of 5-year PFS and OS for all patients were 66.7% (95% CI, 54.2–76.4%) and 84.1% (95% CI, 73.1–90.8%), respectively. When the association of pathological response and survival was evaluated, the percentage 5-year OS of the 20 patients assessed as grade 2 or 3 was 90.0% (95% CI, 65.6–97.4%), whereas that of the 48 patients assessed as grade 0 or 1 was 81.3% (95% CI, 67.1–89.8%).

### Association between radiological response and survival

**Fig. 2:**
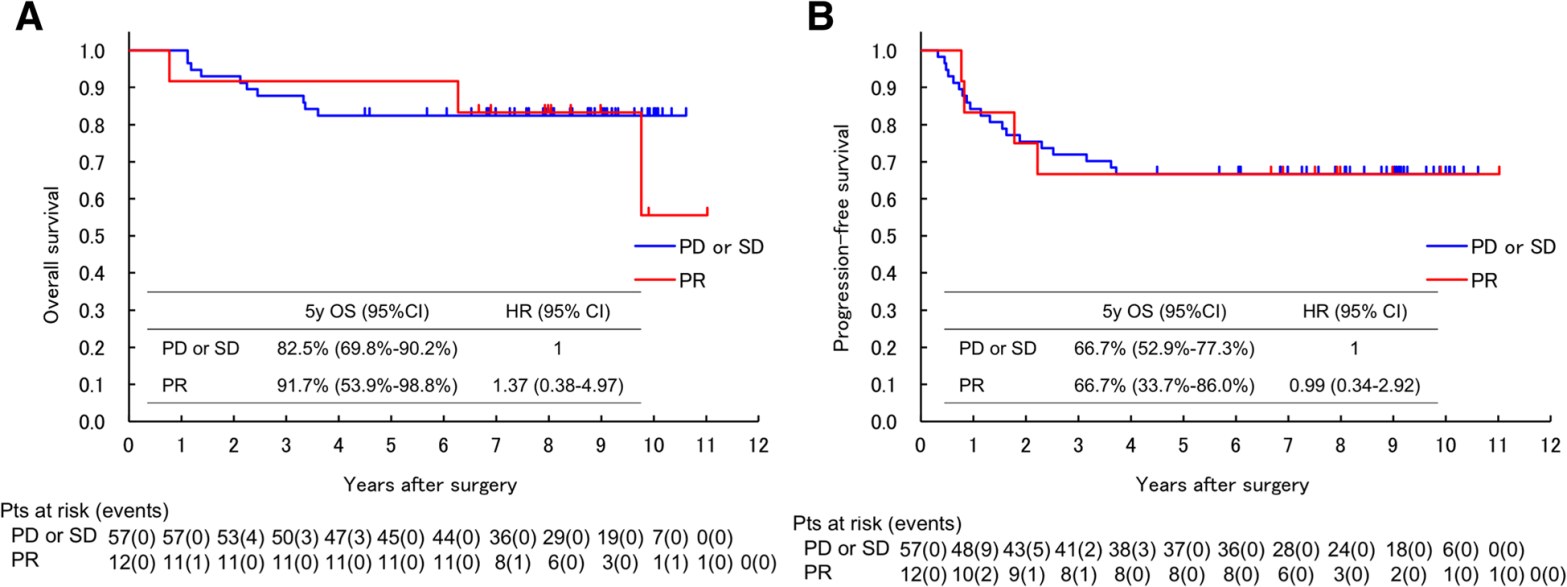
Kaplan-Meier estimates of overall survival (**a**) and progression free survival (**b**) of the patients with STS assessed as PR or SD/PD by RECIST

When the association of radiological response and survival was evaluated, the percentage 5-year OS of the patients assessed by RECIST as PR was 91.7% (95% CI, 53.9–98.8%), whereas that of SD or PD was 82.5% (95% CI, 69.8–90.2%) (Fig. 2a). The difference was not statistically significant (HR 1.37; 95% CI, 0.38–4.97; *p* = 0.63), and calculated C-index for OS was 0.50 (95% CI, 0.15–0.86). The percentage 5-year PFS of the patients assessed by RECIST as PR and SD/PD was 66.7% (95% CI, 33.7–86.0%) and 66.7% (95% CI, 52.9–77.3%), respectively (Fig. 2b). The difference was not statistically significant (HR 0.99; 95% CI, 0.34–2.92; *p* = 0.99), and C-index for PFS was 0.50 (95% CI, 0.23–0.77). As shown in Tables 4 and 5, all other tumor size-based criteria of radiological response did not exhibit significant correlation with OS or PFS of the patients with STS treated by preoperative chemotherapy.

**Table 4:** Correlation between radiological response and OS

	5-year OS of CR/PR/MR (95% CI)	5-year OS of SD/PD (95% CI)	HR (95% CI)	*p* value	C-index (95% CI)
RECIST	91.7% (53.9%–98.8%)	82.5% (69.8%–90.2%)	1.37 (0.38–4.97)	0.63	0.50 (0.15–0.86)
Modified RECIST	93.3% (61.3%–99.0%)	81.5% (68.3%–89.6%)	1.00 (0.28–3.65)	1.00	0.59 (0.25–0.94)
WHO	93.3% (61.3%–99.0%)	81.5% (68.3%–89.6%)	1.00 (0.28–3.65)	1.00	0.59 (0.25–0.94)
JOA	84.0% (62.8%–93.7%)	84.1% (69.5%–92.1%)	1.59 (0.53–4.72)	0.40	0.57 (0.29–0.86)
Modified Choi	81.5% (61.1%–91.8%)	85.7% (70.9%–93.3%)	1.99 (0.67–5.93)	0.21	0.64 (0.39–0.90)

*OS* overall survival, *CR* complete response, *PR* partial response, *MR* minor response, *SD* stable disease, *PD* progressive disease, *HR* hazard ratio, *c-index* concordance index

**Table 5:** Correlation between radiological response and PFS

	5-year PFS of CR/PR/MR (95% CI)	5-year PFS of SD/PD (95% CI)	HR (95% CI)	*p* value	C-index (95% CI)
RECIST	66.7% (33.7%–86.0%)	66.7% (52.9%–77.3%)	0.99 (0.34–2.92)	0.99	0.50 (0.23–0.77)
Modified RECIST	66.7% (37.5%–84.6%)	66.7% (52.4%–77.5%)	1.00 (0.37–2.70)	1.00	0.52 (0.28–0.77)
WHO	66.7% (37.5%–84.6%)	66.7% (52.4%–77.5%)	1.00 (0.37–2.70)	1.00	0.52 (0.28–0.77)
JOA	60.0% (38.4%–76.1%)	70.5% (54.6%–81.6%)	1.41 (0.62–3.21)	0.42	0.56 (0.36–0.77)
Modified Choi	59.3% (38.6%–75.0%)	71.4% (55.2%–82.6%)	1.54 (0.68–3.49)	0.30	0.61 (0.42–0.81)

*CR* complete response, *PR* partial response, *MR* minor response, *SD* stable disease, *PD* progressive disease, *HR* hazard ratio

## Discussion

In this study, we investigated the association of all size-based response criteria with the pathological effects and survival of the patients with operable STS in phase II clinical trial JCOG0304. The results indicated that the size-based response to preoperative chemotherapy was not predictive of histological response or prognosis of the patients with operable STS.

The evaluation of the efficacy of chemotherapy for STS by radiological images has been developed in advanced cases, since the operation is basically not performed for advanced STS and the histological response using the resected specimen cannot be judged. To date, however, there is no consensus which of the criteria is most suitable for evaluation of survival of the patients with advanced STS treated with systemic chemotherapy. Moreover, it has been reported that it was not possible to find a threshold of change in the tumor size associated with survival of advanced STS even if the threshold was varied [23]. Our results were consistent with the past study reporting that there was no association between tumor shrinkage and prognosis in advanced STS.

It is noteworthy that the radiological response criteria have been originally developed for carcinomas, not for sarcomas including STS. In solid tumor cancer, the tumor is mainly composed of cancer cells with scant extracellular matrices. Thus, the death of cancer cells by chemotherapy could directly lead to the reduction of tumor size. The results of the RECIST evaluation of tumor shrinkage in cancer chemotherapy were well associated with prognosis of the cancer patients [24]. In STS, unlike solid cancers, there are rich extracellular matrices around sarcoma cells. Thus, even if sarcoma cells die due to chemotherapy, the size reduction might not be obtained in proportion to the reduction in the number of sarcoma cells since the area of matrices do not change significantly. This might be the reason why the death of sarcoma cells does not always lead to the size reduction of STS and why size-based response criteria could not predict pathological effect or survival of the patients with STS.

In gastrointestinal stromal tumor (GIST), since size reduction of tumor was not an indicator of efficacy of chemotherapy, Choi criteria was developed using the changes not only in tumor size but in density in CT images as a method reflecting the biological state of tumor for the evaluation of efficacy of imatinib on metastatic GIST [12]. It has been also reported that Choi criteria showed better association with PFS and OS in operable STS than RECIST, although the association with pathological response was not analyzed [25]. In the present study, size-based response assessed by modified Choi criteria, where CT-density was not assessed, was not associated with pathological response or survival of the patients with localized STS as well as other response criteria. The biological evaluation using CT density or FDG-PET [26] might be needed to measure the true efficacy of preoperative chemotherapy for operable STS.

The limitations of the present study were as follows: (1) we focused only on the changes in tumor size, and CT density included in the original Choi criteria was not evaluated, (2) the number of patients analyzed and events of OS and PFS were small; therefore, the power and accuracy of the study was not enough to make a conclusion about the surrogacy of radiological response to preoperative chemotherapy for survival of the patients with operable STS. In fact, the power was as low as 20% or less with the number of observed events of 23 to detect the hazard ratio of 1.5 in terms of PFS.

## Conclusion

This is the first report prospectively comparing various size-based response criteria in the preoperative chemotherapy for STS and analyzing the association with both pathological response and survival of the patients with operable STS. The results might suggest that none of the radiological response criteria analyzed demonstrated an association of response to preoperative chemotherapy with pathological response or survival of the patients with operable STS. Thus, further prospective investigation may be needed to develop criteria to evaluate not only tumor shrinkage but biological effects of preoperative chemotherapy for the patients with localized STS.

## Abbreviations

BSTTSG: Bone and Soft Tissue Tumor Study Group
CR: Complete response
CT: Computed tomography
DOX: Doxorubicin
ECOG: Eastern Cooperative Oncology Group
FNCLCC: Federation Nationale des Centers de Lutte Contre le Cancer
GIST: Gastrointestinal stromal tumor
HRs: Hazard ratios
IFM: Ifosfamide
JCOG: Japan Clinical Oncology Group
JOA: Japanese Orthopaedic Association
MR: Minor response
MRI: Magnetic resonance imaging
OS: Overall survival
PD: Progressive disease
PFS: Progression free survival
PR: Partial response
RECIST: Response Evaluation Criteria in Solid Tumors
SD: Stable disease
STS: Soft tissue sarcomas
WHO: World Health Organization
